# 4-Chloro-*N*-(3,5-dimethyl­phen­yl)benzamide

**DOI:** 10.1107/S1600536812007180

**Published:** 2012-02-24

**Authors:** Vinola Z. Rodrigues, B. Thimme Gowda, Viktor Vrábel, Jozef Kožíšek

**Affiliations:** a Department of Chemistry, Mangalore University, Mangalagangotri 574 199, Mangalore, India; b Institute of Physical Chemistry and Chemical Physics, Slovak University of Technology, Radlinského 9, SK-812 37 Bratislava, Slovak Republic

## Abstract

In the mol­ecular structure of the title compound, C_15_H_14_ClNO, the amide group forms dihedral angles of 15.8 (2) and 27.2 (2)°, respectively, with the benzoyl and aniline rings, while the angle between the benzoyl and aniline rings is 11.5 (1)°. The crystal structure is stabilized by N—H⋯O hydrogen bonds, which give rise to infinite chains running along the *c* axis.

## Related literature

For studies, including ours, on the effects of substituents on the structures and other aspects of *N*-(ar­yl)-amides, see: Bowes *et al.* (2003 ▶); Gowda *et al.* (2000 ▶); Rodrigues *et al.* (2011 ▶); Saeed *et al.* (2010 ▶); *N*-(ar­yl)-methane­sulfonamides, see: Gowda *et al.* (2007 ▶); *N*-chloro­aryl­amides, see: Gowda *et al.* (2003 ▶); Jyothi & Gowda (2004 ▶); *N*-bromo­aryl­sulfonamides, see: Usha & Gowda (2006 ▶).
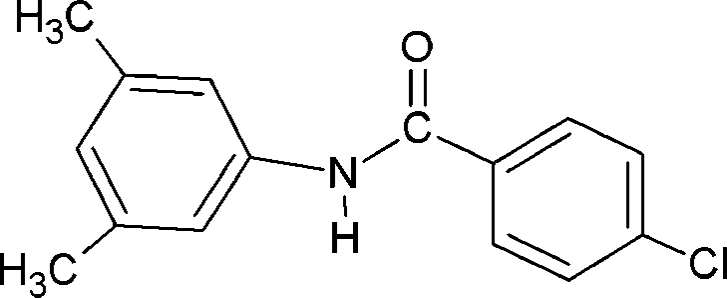


## Experimental

### 

#### Crystal data

C_15_H_14_ClNO
*M* *_r_* = 259.72
Monoclinic, 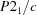

*a* = 14.2763 (7) Å
*b* = 10.7038 (6) Å
*c* = 9.5245 (4) Å
β = 108.087 (5)°
*V* = 1383.52 (12) Å^3^
*Z* = 4
Mo *K*α radiation
μ = 0.26 mm^−1^
*T* = 295 K
0.35 × 0.25 × 0.15 mm

#### Data collection

Oxford Xcalibur CCD diffractometer
Absorption correction: multi-scan (*CrysAlis RED*; Oxford Diffraction, 2009 ▶) *T*_min_ = 0.916, *T*_max_ = 0.958
22077 measured reflections
2433 independent reflections
1542 reflections with *I* > 2σ(*I*)
*R*_int_ = 0.036

#### Refinement

*R*[*F* ^2^ > 2σ(*F* ^2^)] = 0.048
*wR*(*F* ^2^) = 0.152
*S* = 1.03
2433 reflections
169 parameters
1 restraint
H atoms treated by a mixture of independent and constrained refinement
Δρ_max_ = 0.34 e Å^−3^
Δρ_min_ = −0.19 e Å^−3^

### 

Data collection: *CrysAlis CCD* (Oxford Diffraction, 2009 ▶); cell refinement: *CrysAlis CCD*; data reduction: *CrysAlis RED* (Oxford Diffraction, 2009 ▶); program(s) used to solve structure: *SHELXS97* (Sheldrick, 2008 ▶); program(s) used to refine structure: *SHELXL97* (Sheldrick, 2008 ▶); molecular graphics: *DIAMOND* (Brandenburg, 2002 ▶); software used to prepare material for publication: *SHELXL97*, *PLATON* (Spek, 2009 ▶) and *WinGX* (Farrugia, 1999 ▶).

## Supplementary Material

Crystal structure: contains datablock(s) I, global. DOI: 10.1107/S1600536812007180/rk2336sup1.cif

Structure factors: contains datablock(s) I. DOI: 10.1107/S1600536812007180/rk2336Isup2.hkl

Supplementary material file. DOI: 10.1107/S1600536812007180/rk2336Isup3.cml

Additional supplementary materials:  crystallographic information; 3D view; checkCIF report

## Figures and Tables

**Table 1:** Hydrogen-bond geometry (Å, °)

*D*—H⋯*A*	*D*—H	H⋯*A*	*D*⋯*A*	*D*—H⋯*A*
N1—H1⋯O1^i^	0.86 (1)	2.12 (1)	2.948 (2)	163 (2)

Symmetry code: (i) 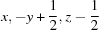.

**Fig. 1.**
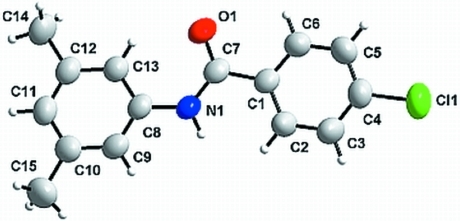
Molecular structure of the title compound showing the atom labelling scheme. Displacement ellipsoids are drawn at the 50% probability level. H atoms are represented as small spheres of arbitrary radius.

**Fig. 2.**
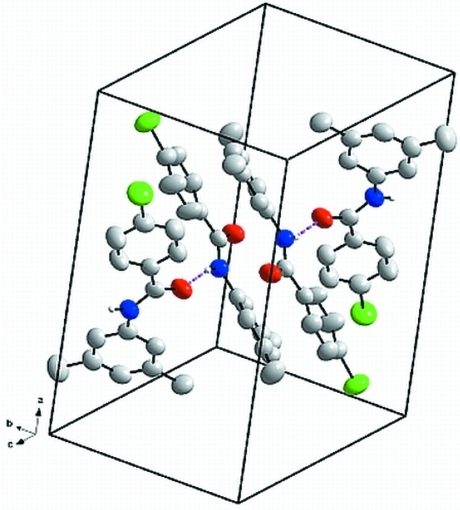
Packing view of the title compound. Molecular chains along c-axis are generated by classical intermolecular N—H···O hydrogen bonds which are shown as dashed lines. Other H atoms have been omitted for clarity.

## supplementary crystallographic information

### Comment

The amide and sulfonamide moieties are the constituents of many biologically important compounds. As part of studies on the substituent effects on the structures and other aspects of *N*-(aryl)-amides (Bowes *et al.*, 2003; Gowda *et al.*, 2000; Rodrigues *et al.*, 2011; Saeed *et al.*, 2010), *N*-(aryl)-methanesulfonamides (Gowda *et al.*, 2007), *N*-chloroarylsulfonamides (Gowda *et al.*, 2003; Jyothi & Gowda, 2004) and *N*-bromoarylsulfonamides (Usha & Gowda, 2006), in the present work, the crystal structure of 4-chloro-*N*-(3,5-dimethylphenyl)benzamide has been determined (Fig. 1).

In the title compound, one of the *m*-methyl groups in the aniline ring is positioned *syn* to the N—H bond, while the other *m*-methyl group is positioned *anti* to the N—H bond, the latter and the C═O bond being *anti* to each other, similar to that observed in 4-chloro-*N*-(3-methylphenyl)benzamide (Rodrigues *et al.*, 2011).

In the title compound, the amide group forms dihedral angles of 15.8 (2)° and 27.2 (2)°, respectively, with the benzoyl and aniline rings, while the angle between the benzoyl and aniline rings is 11.5 (1)°.

In the crystal structure, classical intermolecular N1—H1···O1^i^ hydrogen bonds (Table 1) link the molecules into infinite chains running along the *c*-axis. Symmetry code: (i) *x*, -*y* + 1/2, *z* - 1/2. Part of the crystal structure is shown in Fig. 2.

### Experimental

The title compound was prepared by a method similar to the one described by Rodrigues *et al.* (2011). The purity of the compound was checked by determining its melting point. It was characterized by recording its infrared and NMR spectra.

Plate like colourless single crystals of the title compound used in the X-ray diffraction studies were obtained by slow evaporation of the solvent from its ethanol solution of the compound (0.5 g in about 30 ml of ethanol) at room temperature.

### Refinement

All H atoms bound to carbon were placed in calculated positions with C—H distances of 0.93 Å (C-aromatic), 0.96 Å (C-methyl) and constrained to ride on their parent atoms. The amide H atom was seen in a difference map and refined isotropically. The *U*_iso_(H) values were set at 1.2*U*_eq_(C-aromatic) and 1.5*U*_eq_(C-methyl).

### Crystal data

**Table:**

C_15_H_14_ClNO	*F*(000) = 544
*M**_r_* = 259.72	*D*_x_ = 1.247 Mg m^−^^3^
Monoclinic, *P*2_1_/*c*	Mo *K*α radiation, λ = 0.71073 Å
*a* = 14.2763 (7) Å	Cell parameters from 2433 reflections
*b* = 10.7038 (6) Å	θ = 0.9–1.0°
*c* = 9.5245 (4) Å	µ = 0.26 mm^−^^1^
β = 108.087 (5)°	*T* = 295 K
*V* = 1383.52 (12) Å^3^	Plate, colourless
*Z* = 4	0.35 × 0.25 × 0.15 mm

### Data collection

**Table:**

Oxford Xcalibur CCD diffractometer	2433 independent reflections
Radiation source: fine-focus sealed tube	1542 reflections with *I* > 2σ(*I*)
Graphite monochromator	*R*_int_ = 0.036
Detector resolution: 10.4340 pixels mm^-1^	θ_max_ = 25.0°, θ_min_ = 3.6°
ω scans with κ offsets	*h* = −16→16
Absorption correction: multi-scan (*CrysAlis RED*; Oxford Diffraction, 2009)	*k* = −12→12
*T*_min_ = 0.916, *T*_max_ = 0.958	*l* = −11→11
22077 measured reflections	

### Refinement

**Table:**

Refinement on *F*^2^	Primary atom site location: structure-invariant direct methods
Least-squares matrix: full	Secondary atom site location: difference Fourier map
*R*[*F*^2^ > 2σ(*F*^2^)] = 0.048	Hydrogen site location: inferred from neighbouring sites
*wR*(*F*^2^) = 0.152	H atoms treated by a mixture of independent and constrained refinement
*S* = 1.03	*w* = 1/[σ^2^(*F*_o_^2^) + (0.0866*P*)^2^ + 0.1343*P*] where *P* = (*F*_o_^2^ + 2*F*_c_^2^)/3
2433 reflections	(Δ/σ)_max_ < 0.001
169 parameters	Δρ_max_ = 0.34 e Å^−^^3^
1 restraint	Δρ_min_ = −0.19 e Å^−^^3^

### Special details

**Table:**

Geometry. All s.u.'s (except the s.u. in the dihedral angle between two l.s. planes) are estimated using the full covariance matrix. The cell s.u.'s are taken into account individually in the estimation of s.u.'s in distances, angles and torsion angles; correlations between s.u.'s in cell parameters are only used when they are defined by crystal symmetry. An approximate (isotropic) treatment of cell s.u.'s is used for estimating s.u.'s involving l.s. planes.
Refinement. Refinement of *F*^2^ against ALL reflections. The weighted *R*-factor *wR* and goodness of fit *S* are based on *F*^2^, conventional *R*-factors *R* are based on *F*, with *F* set to zero for negative *F*^2^. The threshold expression of *F*^2^ > σ(*F*^2^) is used only for calculating *R*-factors(gt) *etc*. and is not relevant to the choice of reflections for refinement. *R*-factors based on *F*^2^ are statistically about twice as large as those based on *F*, and *R*-factors based on ALL data will be even larger.

### Fractional atomic coordinates and isotropic or equivalent isotropic displacement parameters (Å^2^)

**Table:**

	*x*	*y*	*z*	*U*_iso_*/*U*_eq_	
C1	0.45950 (16)	0.1593 (2)	0.5588 (2)	0.0589 (6)	
C2	0.41450 (19)	0.2143 (3)	0.4238 (3)	0.0841 (8)	
H2	0.4474	0.2769	0.3902	0.101*	
C3	0.3222 (2)	0.1787 (3)	0.3381 (3)	0.0940 (9)	
H3	0.2935	0.2156	0.2465	0.113*	
C4	0.27271 (18)	0.0882 (3)	0.3885 (3)	0.0733 (7)	
C5	0.3153 (2)	0.0321 (2)	0.5211 (3)	0.0769 (7)	
H5	0.2816	−0.0294	0.5549	0.092*	
C6	0.40845 (18)	0.0672 (2)	0.6046 (3)	0.0717 (7)	
H6	0.4378	0.0276	0.6945	0.086*	
C7	0.55792 (16)	0.1969 (2)	0.6601 (2)	0.0601 (6)	
C8	0.70886 (16)	0.3186 (2)	0.6783 (2)	0.0582 (6)	
C9	0.73635 (18)	0.4271 (2)	0.6217 (2)	0.0664 (6)	
H9	0.6927	0.4638	0.5384	0.080*	
C10	0.82705 (19)	0.4817 (2)	0.6865 (3)	0.0702 (7)	
C11	0.89035 (18)	0.4265 (2)	0.8120 (3)	0.0701 (7)	
H11	0.9513	0.4632	0.8576	0.084*	
C12	0.86517 (17)	0.3179 (2)	0.8711 (2)	0.0643 (6)	
C13	0.77377 (16)	0.2640 (2)	0.8028 (2)	0.0615 (6)	
H13	0.7560	0.1908	0.8408	0.074*	
C14	0.93520 (19)	0.2590 (3)	1.0067 (3)	0.0848 (8)	
H14A	0.9362	0.1702	0.9931	0.127*	
H14B	0.9139	0.2771	1.0907	0.127*	
H14C	1.0002	0.2922	1.0227	0.127*	
C15	0.8581 (2)	0.5971 (3)	0.6211 (3)	0.1037 (10)	
H15A	0.8784	0.6606	0.6957	0.156*	
H15B	0.8037	0.6273	0.5411	0.156*	
H15C	0.9120	0.5768	0.5850	0.156*	
N1	0.61598 (14)	0.26628 (19)	0.60271 (18)	0.0631 (5)	
H1	0.5938 (16)	0.283 (2)	0.5099 (6)	0.070 (7)*	
O1	0.58311 (11)	0.16623 (18)	0.79101 (16)	0.0777 (6)	
Cl1	0.15289 (5)	0.04825 (8)	0.28458 (9)	0.1054 (4)	

### Atomic displacement parameters (Å^2^)

**Table:**

	*U*^11^	*U*^22^	*U*^33^	*U*^12^	*U*^13^	*U*^23^
C1	0.0569 (13)	0.0741 (15)	0.0468 (11)	0.0012 (12)	0.0178 (10)	−0.0037 (11)
C2	0.0613 (15)	0.119 (2)	0.0659 (15)	−0.0208 (15)	0.0113 (12)	0.0201 (15)
C3	0.0700 (17)	0.133 (3)	0.0692 (16)	−0.0179 (17)	0.0069 (14)	0.0241 (16)
C4	0.0609 (15)	0.0874 (18)	0.0697 (15)	−0.0113 (13)	0.0174 (13)	−0.0095 (14)
C5	0.0791 (18)	0.0753 (17)	0.0766 (17)	−0.0167 (14)	0.0247 (14)	0.0008 (14)
C6	0.0728 (16)	0.0820 (18)	0.0591 (14)	−0.0083 (13)	0.0185 (13)	0.0053 (12)
C7	0.0569 (13)	0.0793 (16)	0.0463 (12)	0.0042 (12)	0.0192 (11)	−0.0057 (11)
C8	0.0559 (13)	0.0741 (16)	0.0442 (11)	0.0000 (11)	0.0149 (10)	−0.0091 (11)
C9	0.0721 (16)	0.0753 (16)	0.0491 (12)	−0.0010 (13)	0.0148 (11)	−0.0028 (11)
C10	0.0768 (16)	0.0743 (17)	0.0604 (14)	−0.0110 (13)	0.0227 (13)	−0.0076 (12)
C11	0.0584 (14)	0.0906 (19)	0.0608 (14)	−0.0109 (13)	0.0177 (12)	−0.0154 (13)
C12	0.0556 (13)	0.0816 (17)	0.0549 (13)	0.0019 (12)	0.0161 (11)	−0.0087 (12)
C13	0.0586 (14)	0.0726 (15)	0.0534 (12)	−0.0011 (11)	0.0176 (11)	−0.0034 (11)
C14	0.0606 (16)	0.107 (2)	0.0763 (16)	0.0048 (14)	0.0062 (13)	0.0045 (15)
C15	0.115 (2)	0.098 (2)	0.095 (2)	−0.0299 (19)	0.0264 (18)	0.0059 (17)
N1	0.0596 (11)	0.0856 (14)	0.0414 (10)	−0.0081 (10)	0.0116 (9)	−0.0012 (10)
O1	0.0674 (10)	0.1213 (15)	0.0447 (9)	−0.0051 (9)	0.0177 (8)	0.0041 (9)
Cl1	0.0723 (5)	0.1250 (8)	0.1045 (6)	−0.0281 (4)	0.0065 (4)	−0.0081 (4)

### Geometric parameters (Å, º)

**Table:**

C1—C6	1.375 (3)	C9—C10	1.379 (3)
C1—C2	1.378 (3)	C9—H9	0.9300
C1—C7	1.492 (3)	C10—C11	1.387 (4)
C2—C3	1.371 (4)	C10—C15	1.511 (4)
C2—H2	0.9300	C11—C12	1.387 (3)
C3—C4	1.370 (4)	C11—H11	0.9300
C3—H3	0.9300	C12—C13	1.390 (3)
C4—C5	1.359 (4)	C12—C14	1.504 (3)
C4—Cl1	1.743 (2)	C13—H13	0.9300
C5—C6	1.373 (3)	C14—H14A	0.9600
C5—H5	0.9300	C14—H14B	0.9600
C6—H6	0.9300	C14—H14C	0.9600
C7—O1	1.230 (2)	C15—H15A	0.9600
C7—N1	1.349 (3)	C15—H15B	0.9600
C8—C9	1.387 (3)	C15—H15C	0.9600
C8—C13	1.387 (3)	N1—H1	0.859 (2)
C8—N1	1.414 (3)		
			
C6—C1—C2	117.6 (2)	C9—C10—C11	118.3 (2)
C6—C1—C7	118.2 (2)	C9—C10—C15	121.1 (2)
C2—C1—C7	124.1 (2)	C11—C10—C15	120.5 (2)
C3—C2—C1	121.3 (2)	C12—C11—C10	121.7 (2)
C3—C2—H2	119.3	C12—C11—H11	119.1
C1—C2—H2	119.3	C10—C11—H11	119.1
C4—C3—C2	119.6 (3)	C11—C12—C13	118.8 (2)
C4—C3—H3	120.2	C11—C12—C14	120.9 (2)
C2—C3—H3	120.2	C13—C12—C14	120.4 (2)
C5—C4—C3	120.4 (2)	C8—C13—C12	120.4 (2)
C5—C4—Cl1	119.7 (2)	C8—C13—H13	119.8
C3—C4—Cl1	119.8 (2)	C12—C13—H13	119.8
C4—C5—C6	119.4 (2)	C12—C14—H14A	109.5
C4—C5—H5	120.3	C12—C14—H14B	109.5
C6—C5—H5	120.3	H14A—C14—H14B	109.5
C5—C6—C1	121.7 (2)	C12—C14—H14C	109.5
C5—C6—H6	119.2	H14A—C14—H14C	109.5
C1—C6—H6	119.2	H14B—C14—H14C	109.5
O1—C7—N1	122.3 (2)	C10—C15—H15A	109.5
O1—C7—C1	120.3 (2)	C10—C15—H15B	109.5
N1—C7—C1	117.38 (18)	H15A—C15—H15B	109.5
C9—C8—C13	119.4 (2)	C10—C15—H15C	109.5
C9—C8—N1	117.9 (2)	H15A—C15—H15C	109.5
C13—C8—N1	122.7 (2)	H15B—C15—H15C	109.5
C10—C9—C8	121.4 (2)	C7—N1—C8	127.51 (17)
C10—C9—H9	119.3	C7—N1—H1	117.0 (16)
C8—C9—H9	119.3	C8—N1—H1	115.5 (16)
			
C6—C1—C2—C3	0.0 (4)	N1—C8—C9—C10	−177.95 (19)
C7—C1—C2—C3	−177.5 (2)	C8—C9—C10—C11	−1.0 (3)
C1—C2—C3—C4	1.3 (5)	C8—C9—C10—C15	177.7 (2)
C2—C3—C4—C5	−1.3 (5)	C9—C10—C11—C12	1.1 (4)
C2—C3—C4—Cl1	176.6 (2)	C15—C10—C11—C12	−177.5 (2)
C3—C4—C5—C6	0.1 (4)	C10—C11—C12—C13	−0.4 (3)
Cl1—C4—C5—C6	−177.79 (19)	C10—C11—C12—C14	179.6 (2)
C4—C5—C6—C1	1.1 (4)	C9—C8—C13—C12	0.6 (3)
C2—C1—C6—C5	−1.2 (4)	N1—C8—C13—C12	178.56 (18)
C7—C1—C6—C5	176.5 (2)	C11—C12—C13—C8	−0.4 (3)
C6—C1—C7—O1	−15.0 (3)	C14—C12—C13—C8	179.6 (2)
C2—C1—C7—O1	162.4 (2)	O1—C7—N1—C8	−3.0 (4)
C6—C1—C7—N1	165.9 (2)	C1—C7—N1—C8	176.0 (2)
C2—C1—C7—N1	−16.6 (3)	C9—C8—N1—C7	−151.7 (2)
C13—C8—C9—C10	0.2 (3)	C13—C8—N1—C7	30.3 (3)

### Hydrogen-bond geometry (Å, º)

**Table:**

*D*—H···*A*	*D*—H	H···*A*	*D*···*A*	*D*—H···*A*
N1—H1···O1^i^	0.86 (1)	2.12 (1)	2.948 (2)	163 (2)

Symmetry code: (i) *x*, −*y*+1/2, *z*−1/2.
